# HunFlair2 in a cross-corpus evaluation of biomedical named entity recognition and normalization tools

**DOI:** 10.1093/bioinformatics/btae564

**Published:** 2024-09-20

**Authors:** Mario Sänger, Samuele Garda, Xing David Wang, Leon Weber-Genzel, Pia Droop, Benedikt Fuchs, Alan Akbik, Ulf Leser

**Affiliations:** Department of Computer Science, Humboldt-Universität zu Berlin, Berlin 10099, Germany; Department of Computer Science, Humboldt-Universität zu Berlin, Berlin 10099, Germany; Department of Computer Science, Humboldt-Universität zu Berlin, Berlin 10099, Germany; Center for Information and Language Processing (CIS), Ludwig Maximilian University Munich, München 80539, Germany; Department of Computer Science, Humboldt-Universität zu Berlin, Berlin 10099, Germany; Research Industrial Systems Engineering (RISE) Forschungs-, Entwicklungs- und Großprojektberatung GmbH, Schwechat 2320, Austria; Department of Computer Science, Humboldt-Universität zu Berlin, Berlin 10099, Germany; Department of Computer Science, Humboldt-Universität zu Berlin, Berlin 10099, Germany

## Abstract

**Motivation:**

With the exponential growth of the life sciences literature, biomedical text mining (BTM) has become an essential technology for accelerating the extraction of insights from publications. The identification of entities in texts, such as diseases or genes, and their normalization, i.e. grounding them in knowledge base, are crucial steps in any BTM pipeline to enable information aggregation from multiple documents. However, tools for these two steps are rarely applied in the same context in which they were developed. Instead, they are applied “in the wild,” i.e. on application-dependent text collections from moderately to extremely different from those used for training, varying, e.g. in focus, genre or text type. This raises the question whether the reported performance, usually obtained by training and evaluating on different partitions of the same corpus, can be trusted for downstream applications.

**Results:**

Here, we report on the results of a carefully designed *cross-corpus* benchmark for entity recognition and normalization, where tools were applied systematically to corpora not used during their training. Based on a survey of 28 published systems, we selected five, based on predefined criteria like feature richness and availability, for an in-depth analysis on three publicly available corpora covering four entity types. Our results present a mixed picture and show that cross-corpus performance is significantly lower than the in-corpus performance. HunFlair2, the redesigned and extended successor of the HunFlair tool, showed the best performance on average, being closely followed by PubTator Central. Our results indicate that users of BTM tools should expect a lower performance than the original published one when applying tools in “the wild” and show that further research is necessary for more robust BTM tools.

**Availability and implementation:**

All our models are integrated into the Natural Language Processing (NLP) framework flair: https://github.com/flairNLP/flair. Code to reproduce our results is available at: https://github.com/hu-ner/hunflair2-experiments.

## 1 Introduction

The volume of biomedical literature is expanding at a rapid pace, with public repositories like PubMed housing over 30 million publication abstracts. A major challenge lies in the high-quality extraction of relevant information from this ever-growing body of literature, a task that no human can feasibly accomplish, thus requiring support from computer-assisted methods.

**Figure 1.**
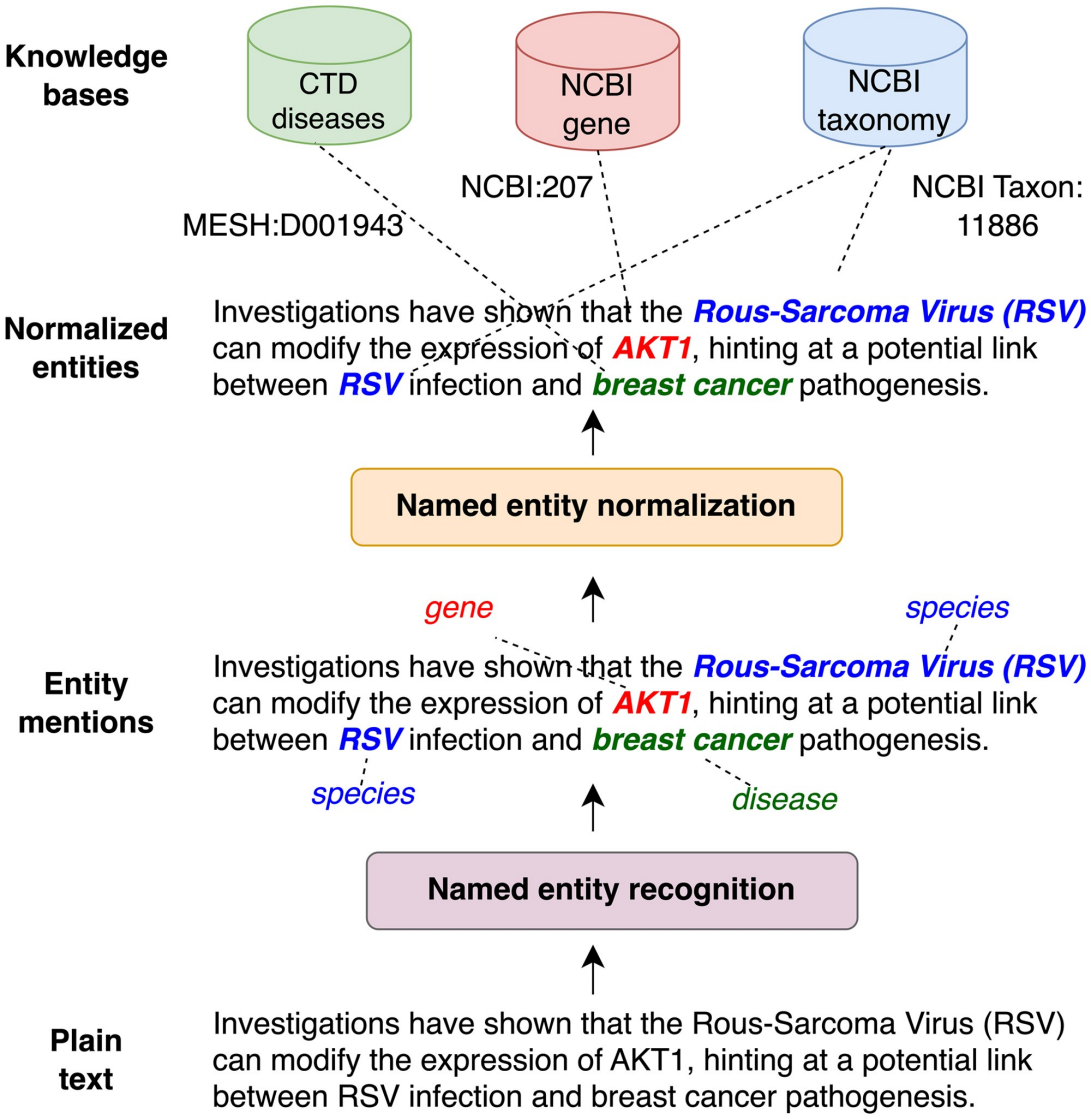
Illustration of named entity extraction. First entity mentions are identified (NER) and then mapped to standard identifiers in a knowledge base (NEN).

A crucial step in such pipelines is the extraction of biomedical entities (such as genes/proteins and diseases) as it is a prerequisite for further processing steps, like relation extraction (Weber *et al.* 2022), knowledge base (KB) completion (Sänger and Leser 2021) or pathway curation (Weber *et al.* 2020). As shown in Fig. 1, this typically involves two steps: (i) named entity recognition (NER) and (ii) named entity normalization (NEN) (a.k.a entity linking or entity disambiguation. We refer to their combination as extraction). NER identifies and classifies entities discussed in a given document. However, different documents may use different names (synonyms) to refer to the same biomedical concept. For instance, “tumor protein p53” or “tumor suppressor p53” are both *valid* names for the gene “TP53” (NCBI Gene: 7157). The same mention can refer as well to different entities (homonyms), e.g. “RSV” can be “Rous-Sarcoma-Virus” or “Respiratory syncytial virus” depending on context. Entity normalization addresses the issues of synonyms and homonyms by mapping mentions found by NER to a KB identifier. This process ensures that all entity mentions are recognized as referring to the concept, regardless of how they are expressed in the text, allowing to aggregate and compare information across different documents.

Over the last two decades, several studies investigated biomedical NER and NEN. Of the many research prototypes, some have been consolidated into mature and easy-to-install or use *tools* that end users can apply directly for their specific needs (Wei *et al.* 2019, Weber *et al.* 2021, Zhang *et al.* 2021, inter-alia). These tools are commonly deployed “in the wild,” i.e. to custom text collections with specific focus (e.g. cancer or genetic disorders), entity distribution (gene-focused molecular biology or disease-focused clinical trials), genre (publication, patent, report) and text type (abstract, full text, user-generated content). The tools, however, were originally trained and evaluated on a single or few gold standard corpora, each having its own specific characteristics (focus, entity distribution, etc.). The mismatch between these two settings, i.e. training/evaluation versus downstream deployment, raises the question whether the performance in the first can be trusted to estimate the one achievable in the second. As named entity extraction is the cornerstone of several applications, e.g. for relation and event extraction (Wang *et al.* 2020), the issue has a direct and critical impact on downstream information extraction pipelines.

To better quantify the impact of this issue, previous work proposed to use a *cross-corpus* evaluation, i.e. training models on one corpus and evaluating on a different one (Galea *et al.* 2018). For instance, Giorgi and Bader (2020) show that the performance of neural networks for NER drops by an average of 31.16% *F*1 when tested on a corpus different from the one used for training. Previous studies, however, present a few limitations: First, existing benchmarking studies, both in- and cross-corpus, focus primarily either on recognition (Song *et al.* 2021, Su *et al.* 2022) or normalization (French and McInnes 2022, Garda *et al.* 2023) but do not provide results for named entity extraction, i.e. end-to-end NER and NEN. Secondly, many of them do not account for the latest technologies and models (Wang *et al.* 2018) like pretrained language models.

In this study, we address these limitations and present the first cross-corpus evaluation of state-of-the-art tools for named entity extraction (end-to-end NER and NEN) in biomedical text mining (BTM) to provide an in-depth analysis of their results, highlighting current limits and areas for improvement. After an extensive literature review that identified 28 publications, we select from these five mature tools based on predefined criteria: (C1) supporting both NER and NEN, (C2) integrating recent improvements (e.g. transformers), (C3) extracting the most common entities (genes, disease, chemicals, and species), and (C4) requiring no further licenses (e.g. Unified Medical Language System (UMLS) license). The tools are: BERN2 (Mujeen *et al.* 2022), PubTator Central (PTC; Wei *et al.* 2019), SciSpacy (Neumann *et al.* 2019), and bent (Ruas *et al.* 2023). In the comparison, we include as well HunFlair2, our novel and extended version of HunFlair (Weber *et al.* 2021).

We performed extensive experiments on three corpora covering four entity types and diverse text forms (abstracts, full text, and figure captions). The corpora were explicitly selected such that none of the tools (according to their documentation) used them for training. Our results show stark performance variations among tools w.r.t. entity types and corpus, with differences of up to 54 percentage points (pp) when comparing scores to those of previously published in-corpus results. The overall most robust tool was HunFlair2, reaching an average *F*1-score of 59.97% and the best results in two (chemicals and diseases) of four entity types. PTC scores very close second best on average and best in the other two types (genes and species). Our study highlights the need of further research toward more robust BTM tools to account for change in biomedical subdomains and text types in downstream applications.

## 2 Materials and methods

We now describe the selected tools and the evaluation framework used to assess their “in the wild” performance, using a *cross-corpus* evaluation protocol. Intuitively, by applying them to diverse texts not part of their training, we assess their robustness w.r.t. shifts in central characteristics like text types, genres, or annotation guidelines. We stress that our evaluation strictly focuses on *tools* capable of both *NER* and *NEN*. For recent benchmarks on NER and NEN, we refer the reader to Keloth *et al.* (2024) and Garda *et al.* (2023), respectively.

### 2.1 Tools

We first survey existing BTM tools via Google Scholar. We define a tool to be a piece of software, i.e. (a) either accessible online via an application programming interface (API) or installable locally, and (b) usable off-the-shelf requiring minimal configurations by nonexperts. This results in an initial list of 28 candidates. From these, we selected tools for in-depth analysis based on the following criteria. The tool must:

- C1: support both NER and NEN.
- C2: use machine-learning-based models (at least for NER).
- C3: extract at least genes, diseases, chemicals, and species.
- C4: not require additional licenses (e.g. commercial or UMLS license).

This results in the selection of the following tools (see Table 1 for an overview): BERN2 (Mujeen *et al.* 2022), bent (Ruas *et al.* 2020, 2023), PTC (Wei *et al.* 2019), and SciSpacy (Neumann *et al.* 2019). An overview of all surveyed tools and details on the selected ones (training corpora, architecture, etc.) can be found in Supplementary Data A. We also provide there an in-depth analysis for HunFlair2, our updated version of HunFlair (Weber *et al.* 2021) which we first introduce here.

**Table 1.** Overview of the tools selected for our evaluation.

Tool	API	Ge	Sp	Di	Ch	Cl	Va	NER	NEN	Pub. year	Last update	Citations
PubTator Central	REST/	✔	✔	✔	✔	✔	✔	ML/NN	RB	2019	–	315
Wei *et al.* (2019)	Tools											
BERN2	REST/	✔	✔	✔	✔	✔	✔	NN	RB/NN	2022	11/2023	46
Mujeen *et al.* (2022)	Python											
SciSpacy	Python	(✔)	✔	✔	✔	✔	(✔)	NN	RB	2019	10/2023	635
Neumann *et al.* (2019)												
bent	Python	✔	✔	✔	✔	✔	(✔)	NN	RB	2020	12/2023	13
Ruas *et al.* (2020, 2023)												
HunFlair2	Python	✔	✔	✔	✔	✔		NN	RB/NN	2021	01/2024	83
–												

Supported entity types: genes (Ge), species (Sp), disease (Di), chemical (Ch), cell line (Cl), and variant (Va). We use ✔ if that the tool supports both NER and NEN for the entity type, (✔) if only NER. Methods are categorized as rule-based (“RB”), machine-learning-based (“ML”), or neural-network-based (“NN”). Last update highlights are based on the public code repository while citation counts are taken from Google Scholar on 10 January 2024.

**HunFlair2** adds to HunFlair (a) support for normalization and (b) an update to the recognition component by replacing the recurrent neural network-based character language models with the LinkBERT pretrained language model (Yasunaga *et al.* 2022). HunFlair2 supports the extraction of five biomedical entity types: cell lines, chemicals, diseases, genes, and species. For NER, HunFlair2 employs a single model that identifies at once mentions of any entity type instead of training a separate model for each one, inspired by the all-in-one NER approach (AIONER) of Luo *et al.* (2023). Differently from Luo *et al.* (2023), we use a natural language template in imperative mode to specify which entity types to extract, e.g. “[Tag genes] *<input-example>*” to extract genes only and “[Tag chemicals, diseases and genes] *<input-example>*” for multiple entity types at once. Output labels are assigned using the standard IOB labels, with *B-<entity type>* and *I-<entity type>* denoting a particular type. We omit the use of *O-<entity type>* labels as proposed by Luo *et al.* (2023) and use standard multi-task learning with *O* labels. HunFlair2’s NER model is trained on: NLM Gene (Islamaj *et al.* 2021b), GNormPlus (Wei *et al.* 2015), Linneaus (Gerner *et al.* 2010), S800 (Pafilis *et al.* 2013), NLM Chem (Islamaj *et al.* 2021a), SCAI Chemical (Kolárik *et al.* 2008), NCBI Disease (Leaman and Lu 2016), SCAI disease (Gurulingappa *et al.* 2010) and BioRED (Luo *et al.* 2022). Overall, HunFlair2 improves 2.02 pp over HunFlair across entity types and corpora (see Supplementary Data D). Similar to BERN2, the normalization component uses pretrained BioSyn models (Sung *et al.* 2020) for gene, disease and chemical normalization which link to NCBI Gene (Brown *et al.* 2015), Comparative Toxicogenomics Database (CTD) Diseases (a.k.a. MEDIC, a subset of MeSH and OMIM) and CTD Chemicals (a subset of MESH; Davis *et al.* 2023), respectively. We note that, as in BERN2, the gene model was trained on the BC2GN corpus (Morgan *et al.* 2008), and links exclusively to the human subset of the NCBI Gene (Brown *et al.* 2015). For species, since there is no available BioSyn model, we rely instead on SapBERT (Liu *et al.* 2021), a BioBERT model pretrained on UMLS, which includes NCBI Taxonomy (Scott 2012), the species ontology to which we link.

### 2.2 Corpora

Designing a cross-corpus benchmark for entity extraction poses unique challenges in terms of data selection, as corpora must meet the following conditions: (a) they have not been used to train (train or development split) any of the tools, (b) they contain both NER and NEN annotations and (c) their entity types are normalized to KBs supported by all tools. To ensure these conditions, we select the following corpora: BioID (Arighi *et al.* 2017), MedMentions (Mohan and Li 2019) (We use the subset ST21pv targeting information retrieval.), and tmVar (v3; Wei *et al.* 2022). The corpora present annotations covering four entity types: genes, species, disease, and chemicals, which are linked to NCBI Gene, NCBI Taxonomy, CTD Diseases, and CTD Chemicals, respectively. We note that in MedMentions annotated spans are linked to UMLS (Bodenreider 2004). However, as UMLS provides cross-reference tables with MeSH and OMIM, we are able to map its annotations to the CTD dictionaries and the entity type as either disease or chemical based on the CTD vocabulary to which the identifier has been successfully mapped (We use UMLS 2017AA, the one used to create MedMentions.). We analyze the coverage of this mapping strategy and its impacts on result interpretation in Section 4.4. In Table 2, we present an overview of the corpora (see Supplementary Data B for a detailed description), which we access via BigBio (Fries *et al.* 2022), a community library of biomedical NLP corpora. As none of the corpora is used by any of the tools, we always use the entire corpus for evaluation rather than their predefined test splits.

**Table 2.** Overview of the corpora selected for our evaluation.

				Chemical		Disease		Gene		Species	
Corpus	Text type	Documents	Tokens	Mentions	Uniq.	Mentions	Uniq.	Mentions	Uniq.	Mentions	Uniq.
BioID (Arighi *et al.* 2017)	Figure captions	13 697	708 913	-		-		-		7949	149
				-		-		-		(*NCBI Taxonomy*)	
MedMentions (Mohan and Li 2019)	Abstract	4392	1 012 453	19 199	2531	19 298	1694	-		-	
				(*CTD Chemicals*)^a^		(*CTD Diseases*)^a^		-		-	
tmVar (v3; Wei *et al.* 2022)	Abstract	500	119 066	-		-		4059	677	-	
								(*NCBI Gene*)			

a We show: total number of entity mentions, the number of unique entities, and the KB used for normalization (in parenthesis). We only report the entity type used our evaluation. MedMentions uses UMLS for normalization. For our evaluation, we map identifiers to the CTD vocabularies with UMLS cross-reference tables (see Section 2).

### 2.3 Metrics

For all tools, we report *mention-level* micro (average over mentions) *F*1-score. As entity extraction accounts for both recognition and normalization, predictions and gold labels are triplets: start and end offset of the mention boundary and KB identifier. Following Weber *et al.* (2021), the predicted mention boundaries are considered correct if they differ by only one character either at the beginning or the end. This is to account for different handling of special characters by different tools, which may result in minor span differences (see Supplementary Data E1). A predicted triplet is a true positive if both the mention boundaries and the KB identifier are correct. As in Garda *et al.* (2023), for mentions with multiple normalizations, e.g. composite mentions (“breast and squamous cell neoplasms”), we consider the predicted KB identifier correct if it is equal to any of the gold ones (We note, however, that these cases are rare: 90 out of 4059 in tmVar v3, 3 out of 7949 in BioID and none in MedMentions.). To address the incompleteness of the UMLS-MESH cross-reference tables while creating the gold standard for chemicals and diseases, we deviate from this general framework in these two scenarios and ignore predictions that exactly match nonmappable entities; i.e. we count them neither as false nor true positives. We treat each annotated entity having a semantic category linked to *Chemicals & Drugs* and *Disorders* as potential chemicals and diseases, respectively (see https://lhncbc.nlm.nih.gov/ii/tools/MetaMap/Docs/SemGroups_2018.txt).

## 3 Results

In Table 3, we report the results (micro *F*1) of our cross-corpus entity extraction evaluation (see Table 7 in Supplementary Data for precision and recall). First, we note that our results confirm previous findings (Giorgi and Bader 2020, Weber *et al.* 2021): when applied to texts different from those used during training, the performance to be expected from current tools is significantly reduced compared to published results (see Section 4.1 for an in-depth discussion). Unlike previous studies, which considered only entity recognition, the drop in performance is even larger, which can be explained by the increased complexity of the task (NER and NEN). The overall best-performing model is HunFlair2, with PTC being second, both having considerably higher average performance than the other three competitors.

**Table 3.** Mention-level micro *F*1 for end-to-end entity recognition and normalization in a cross-corpus setting.

	BERN2	HunFlair2	PTC	SciSpacy	bent
*Chemical*					
MedMentions	43.37 (34.68^a^)	**54.11**	32.05	36.32	42.57
*Disease*					
MedMentions	48.54	**59.37**	42.03	41.85	47.20
*Gene*					
tmVar (v3)	43.96	76.75	**86.02**	−^b^	0.54
*Species*					
BioID	14.35	49.66	**58.90**	37.14	10.35
Avg	37.56 (35.37^a^)	**59.97**	54.75	38.43	25.16

a Results including ChEBI annotations: see Section 4.1.

b SciSpacy does not support gene normalization.

Bold values highlight the highest score per data set/row.

The instances where these two models stand out are the gene and species corpora. For genes, PTC outperforms all models, while HunFlair2 is second. PubTator’s advantage can be explained by its highly specialized GNormPlus system usage. Secondly, as HunFlair2 does not use context information for linking, it cannot effectively handle intra-species gene ambiguity, e.g. “TPO” can be either the human gene 7173 (“thyroid peroxidase”) or 7066 (“thrombopoietin”) depending on the context. Though BERN2 also uses GNormPlus, its performance is notably lower than PubTator’s. This is attributable to its NER component, which introduces many false positives. SciSpacy achieves a subpar performance for chemicals and diseases while scoring third best for species. Finally, we note bent’s exceptionally low score on genes. By inspecting its predictions, we find that this is due to the tool consistently predicting genes of species that are not human. For instance, all mentions of “BRCA1,” instead of being linked to the human gene (672), are linked to the Capuchin monkey (108289781). As 96% of mentions in tmVar (v3) are human genes, this drastically impacts bent’s results. Regarding species, the leading cause for the low performance of BERN and bent are subtle differences in the KB identifiers, primarily for mentions of the mouse concept. Mouse is one of the organisms most frequently mentioned in biomedical publications. In BioID, its mentions are linked to NCBI Taxonomy 10090 “house mouse.” While both PTC and HunFlair2 also return 10090, bent links mentions of mouse to NCBI Taxonomy 10088 (“mouse genus”), while BERN2 to 10095 (“unspecified mouse”), causing a drastic drop in performance. For diseases, we see that differences are not as pronounced with almost all tools achieving >40% *F*1-score, where we attribute HunFlair2’s advantage to its superior NER performance (see below). Interestingly, BERN2 comes as a close second. We hypothesize this is due to the better performance of its neural normalization components for diseases and chemicals.

## 4 Discussion

### 4.1 Cross-corpus versus in-corpus evaluation

The effectiveness of named entity extraction tools is most often measured in an in-corpus setting, i.e. training and test data come from the same source. However, in downstream applications, such consistency cannot be assumed, as tools need to process documents widely different from those used in their training. Therefore, reported performance does not represent how tools generalize to the downstream setting, most likely overestimating their ability to handle variations. A cross-corpus evaluation (Galea *et al.* 2018) allows to (partly) overcome this limitation by training and evaluating on *different* corpora, thus accounting for variations in text collections (focus, concept definitions, etc.). For instance, as shown in Table 4, we see that both tools report drastically higher scores for an in-corpus NEN evaluation compared to the corresponding cross-corpus one (The only exception is gene extraction with PTC where cross-corpus performance is higher than the in-corpus one. The difference can be attributed to the fact that in tmVar v3 ∼96% of the genes are human, while these are only 48% in NLM-Gene, thus being significantly more challenging due to multi-species gene ambiguity, Wei and Kao (2011).).

**Table 4.** We show the differences between in- and cross-corpus performances based on results reported by PubTator and BERN2.

Tool	In-corpus	Cross-corpus
BERN2		
*Chemical*	96.60^a^ (*BC5CDR*)	43.37 (*MedMentions*)
*Disease*	93.90^a^ (*BC5CDR*)	48.54 (*MedMentions*)
*Gene*	95.90^a^ (*BC2GM*)	43.96 (*tmVar v3*)
PTC		
*Chemical*	77.20 (*NLM-Chem*)	32.05 (*MedMentions*)
*Disease*	80.70 (*NCBI-Disease*)	42.03 (*MedMentions*)
*Gene*	72.70 (*NLM-Gene*)	86.02 (*tmVar v3*)

In-corpus (evaluation corpus in brackets) results are only for NEN (gold mentions) and not (as in our study) the end-to-end NER and NEN. Since the tools leverage different corpora for evaluation results are not directly comparable. They are, however, indicator of the *magnitude* of difference between in- and cross-corpus performance.

a Result represents accuracy scores.

Establishing a robust and fair cross-corpus evaluation for entity extraction presents, however, its own challenges. A major issue is the difference across corpora w.r.t. mention boundaries, stemming from differences in the annotation guidelines. This can lead to a conflict between training and test annotations, e.g. if one guideline allows composite mentions and the other not (“breast and ovarian cancer” versus “breast” and “ovarian cancer”). As many applications do not require mention boundaries, e.g. semantic indexing (Leaman *et al.* 2023), it is common to measure the document-level performance. Under this setting, we find that results for all tools improve up to 8 pp (see Supplementary Data F3 for details). Related to this issue is the difference in KBs used for normalization. Different corpora and tools often use different KBs; hence, a mapping of identifiers is necessary for a fair comparison, e.g. UMLS in our case (see Section 2). However, cross-reference tables to map concepts between ontologies are not always available. For instance, BERN2 normalizes chemicals both to CTD Chemicals (65%) and ChEBI (35%). However, to the best of our knowledge, no mapping is available between the two. We were therefore forced to modify the BERN2 installation to only use CTD, ignoring many of its normalization results. Finally, minimal variations in the normalization choices can introduce substantial differences in results, as exemplified by the “mouse” case reported in Section 3. This can be mitigated by using evaluation metrics that take into account the KB hierarchy, e.g. the one introduced by Kosmopoulos *et al.* (2015), which considers the lowest common ancestor for the evaluation, penalizing predictions according to the KB hierarchy. This is, however, limited to KBs that are structured hierarchically, which is not always the case (e.g. NCBI Gene). The scarcity of corpora with linking annotations further complicates a cross-corpus evaluations. For instance, for cell lines, there are no other corpora than BioID (Arighi *et al.* 2017) and JNLPBA (Collier and Kim 2004), both of which are used during training by at least one of the evaluated tools. Secondly, we are restricted to corpora which link to ontologies supported by the evaluated tools. Finally, we note that can assess the tools’ performance only on one corpus per entity type, limiting our expressiveness of our evaluation. These limitations must be taken into account when interpreting the results.

### 4.2 Entity distribution

Corpora are often designed for specific subdomains or applications, e.g. the plant-disease-relation corpus (PDR) (Cho *et al.* 2017) focuses on plants, while BioNLP2013-CG (Pyysalo *et al.* 2013) does on cancer genetics. Consequently, they often present imbalanced entity distributions, with few highly frequent entities and a long tail of rarely occurring ones. For instance, as shown in Table 5, in BioID, the most frequent species is NCBI Taxonomy 10090, accounting for more than half of all mentions in the corpus. This raises the question of how much the performance can be attributed to correctly extracting the most frequent entities. For this, we compute macro-average *F*1-scores (see Supplementary Data C for details) as well. The results show strong performance degradation in all tools across all entities, most notably for species, which is consistent with the entity distribution in Table 5. We refer the reader to Table 8 in Supplementary Data F2 for complete results (and Table 6 in Supplementary Data E3 for the NER-only equivalent).

**Table 5.** Distribution statistics of the top three species entities in BioID (right) with the corresponding three most frequent mentions.

Entity	Count (%)	Mention	Count (%)
NCBI Taxonomy: 10090	4002 (50.35%)	Mice	2923 (36.77%)
		Mouse	396 (4.98%)
		Mice	64 (0.81%)
NCBI Taxonomy: 9606	688 (8.66%)	Human	228 (2.87%)
		Patients	163 (2.05%)
		Patient	119 (1.5%)
NCBI Taxonomy: 7227	298 (3.75%)	Flies	134 (1.69%)
		Larvae	53 (0.67%)
		Fly	18 (0.23%)

In addition to entity distribution biases in single corpora, the entities seen during training influence the tools’ performance. We analyze the overlaps of occurring entities in the training and (cross-corpus) test corpora to get insights into this factor. To quantify the overlaps, we rely on the redundancy and zero-shot values introduced by Ferré and Langlais (2023). The former measures the size of the intersection between unique train and test concepts divided by the total number of unique train concepts. The latter represents the ratio of unique test concepts not occurring in the train set (A redundancy score close to zero and a zero-shot score close to one indicate highly different train and test datasets.). We compute both scores for the gene and disease components of HunFlair2. The results of our analysis can be found in Table 6. For training HunFlair2’s gene recognition, GNormPlus, NLM Gene, and BioRED are used. Even though the redundancy between the train sets and the test dataset (i.e. tmvar v3) is low (0.08), the reported cross-corpus performance reaches a compelling *F*1-score of 76.75%. Concerning zero-shot instances, a value of 0.30 indicates that most genes have already been seen in the training dataset, making it easier for HunFlair2 to detect them. In the case of diseases, HunFlair2 was trained on NCBI Disease and BioRED (Note, we exclude SCAI diseases from this analysis as it includes no KB identifiers in its annotations.). In this scenario, we observe stark performance drops in our cross-corpus evaluation using MedMentions as test corpus (see Table 3). Although the redundancy between train and test corpora is much higher for diseases than for genes, the zero-shot ratio increases strongly, making it much harder for the extraction models to memorize already-seen entities.

**Table 6.** Dataset similarities between train and test corpora used by HunFlair.

Entity	Train	Test	Redundancy	Zero-shot
Gene	GNorm Plus + NLM Gene + BioRED.	tmVar v3	0.08	0.30
Disease	NCBI Disease + BioRED.	MedMentions	0.49	0.64

### 4.3 NER performance

**Figure 2.**
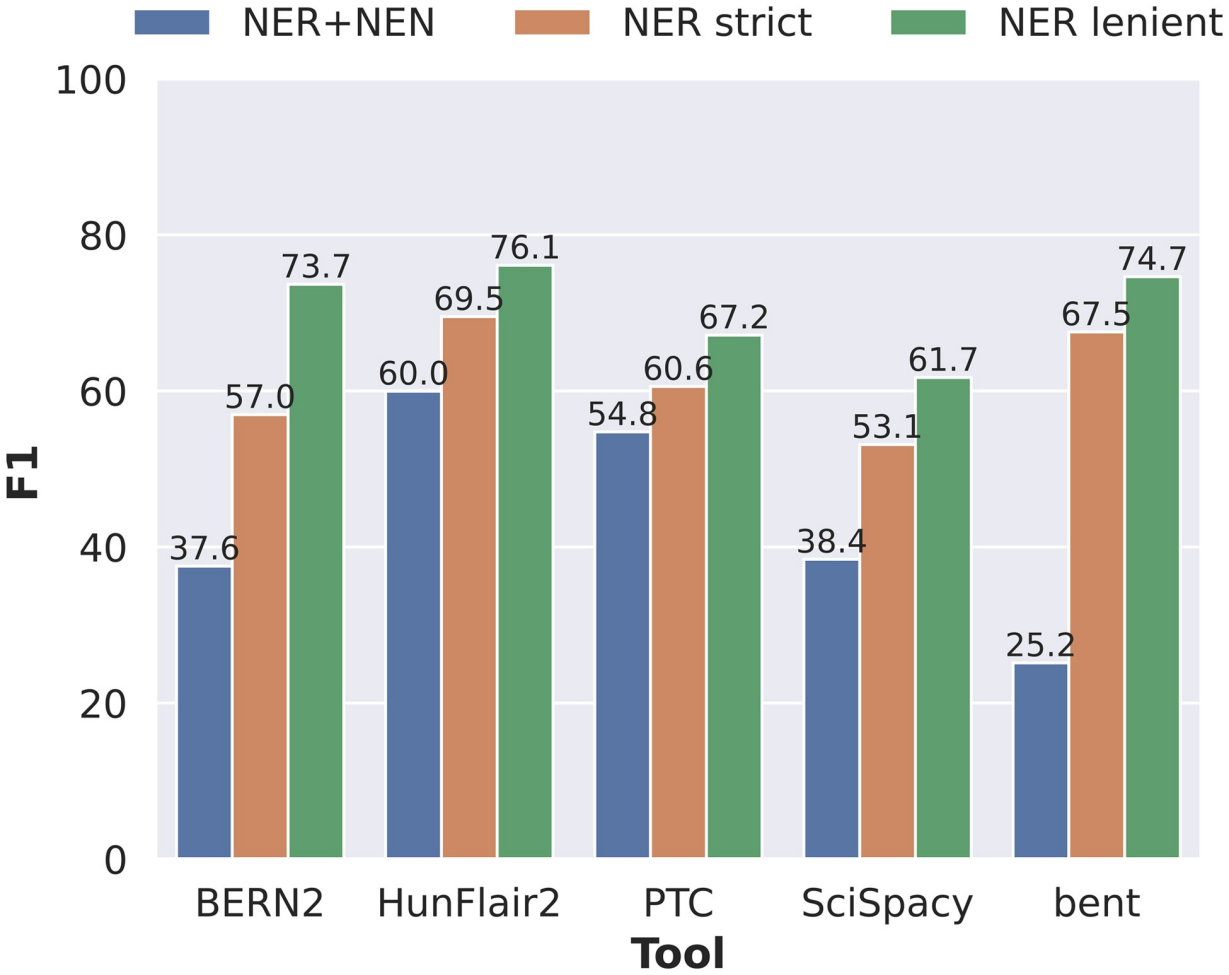
Performance comparison of the five tools concerning end-to-end NER+NEN, NER strict, and NER lenient evaluation settings, where each prediction is counted as a true positive if it is either a substring or a superstring of the gold standard entity mention.

We examined the performance of the NER step separately to determine how much influence each step of the BTM pipeline has exactly on the overall performance and how much of the errors can be attributed to error propagation from the NER step. To this end, we compared the NER+NEN results to two “NER only” settings in Fig. 2. In the strict setting, detected entities that differ at most one character are still counted as true positives. In the lenient setting, detected entities that differ by at most one character and/or are a superstring or substring of a ground truth entity are counted as true positives.

HunFlair2 and PTC showed the most consistent performances across the three settings, indicating relatively robust performances for both the NER and NEN steps. The other three tools show much larger discrepancies between NER performances and joint NER+NEN results, indicating weaknesses in the normalization components. The largest drop between NER and joint NER+NEN is found in bent where performance in NER strict comes only second to HunFlair2 by a gap of 2.0 pp. When measuring NER+NEN, the *F*1-score then drops down to 25.2%. BERN2 is the only tool that shows significant discrepancy from NER lenient to NER strict evaluation, with the *F*1-score decreasing by 16.7 pp from 73.7% to 57.0%. A closer inspection of the BERN2 predictions reveals that the performance differences mostly stem from the handling of the token “gene” in gene entities. When the term “gene” immediately follows after a gene mention, e.g. in “AKT-1 gene,” BERN2 often annotates the term “gene” as part of its prediction, whereas the gold standard corpus does not. A detailed overview of NER performances split by corpus and entity types is given in Supplementary Data E2.

### 4.4 Reference ontologies

Creators of annotated corpora must decide, for each entity type, which ontology to use as the target for the normalization step (i.e. for NEN). Often, the entity annotation is already defined by a chosen ontology to have a clear definition of what to annotate and what not, i.e. already during NER. Furthermore, developers of NEN tools decide on which corpora they use for training, which implicitly also is a decision on which ontologies they can map to. To compare tools on corpora, which were designed for different ontologies for a given entity type, some form of ontology mapping has to be applied. Research in this field is rich (Euzenat *et al.* 2013) and often also addresses the issue of properly scoring hypernyms and hyponyms, i.e. cases where normalization yields a concept ID that is a generalization or specialization of the annotated concept ID (Lord *et al.* 2003, Groth *et al.* 2008).

In this work, we restricted our evaluation to mappings readily provided with an ontology, for instance, the MeSH mappings for UMLS terms defined within UMLS, irrespective of their quality or coverage. This ensures that our results can be compared easily to past and future NEN evaluations with these ontologies, which would not be the case when custom-computed mappings are introduced. However, the applied mapping strategy introduces a bias toward specific systems. For example, it favors tools that predict coarse-grained MESH concepts instead of ones adhering to more fine-granular UMLS concepts. To account for this situation, we skip all predictions that exactly match nonmappable entities in our evaluation. To gain further insight into the impact of the mapping strategy, we computed the number of nonmappable entities mentioned in MedMentions to quantify this issue. We found that only 55.1% of the 35 014 diseases and 50.5% of the 38 037 chemical mentions could be mapped to MESH, limiting our evaluation. We list the 50 most frequent nonmappable entities, representing 30.4%/49.7% of all nonmappable disease/chemical mentions, in Supplementary Data G. The analysis shows that the top entities refer to rather general concepts such as *pharmacologic substance* (C1254351), *proteins* (C0033684), *finding* (C0243095), or *diagnosis* (C0011900). These investigations highlight that further research is strongly needed to harmonize existing and create additional NEN datasets, enabling a more robust assessment.

## 5 Conclusion

In this work, we reviewed 28 recent tools designed for extracting biomedical named entities from unstructured text regarding their maturity and ease of usage for downstream applications. We selected five tools, namely BERN2, bent, HunFlair2, PTC, and SciSpacy, for a detailed examination and assessed their performance on three different corpora, encompassing four types of entities, following a cross-corpus approach. Our experiments highlight that the performance of the tools varies considerably across corpora and entity types. Additionally, we found strong performance drops compared to the published in-corpus results. In-depth prediction analyses revealed that the tools demonstrate strong performance when identifying highly researched entities; however, they face challenges in accurately identifying concepts that rarely occur in the literature. In conclusion, our results illustrate that further research is needed on the generalization capabilities of named entity extraction tools to facilitate their seamless application to diverse biomedical subdomains and text types. In addition, our study highlights the crucial need to create additional NEN datasets and harmonize existing ones to foster a more effective evaluation of the available tools.

## Supplementary Material

btae564_Supplementary_Data

## Data Availability

The data underlying this article are available in hunflair2-experiments GitHub repository at https://github.com/hu-ner/hunflair2-experiments, and can be accessed using commit hash c9725df8ce2a42a00ff468f183406119f50397fd.
