# Supplementary material for: HunFlair2 in a cross-corpus evaluation of biomedical named entity recognition and normalization tools
Source: Bioinformatics. 2024 Sep 20;40(10):btae564. doi: 10.1093/bioinformatics/btae564 (PMC11453098; doi:10.1093/bioinformatics/btae564)
Supplement: btae564_Supplementary_Data [file btae564_supplementary_data.pdf]

Journal Title Here, 2022, 1–11
doi: DOI HERE
Advance Access Publication Date: Day Month Year
Paper

HunFlair2 in a cross-corpus evaluation of biomedical named entity recognition and normalization tools

Mario Sänger,[1,*,†] Samuele Garda,[1,†] Xing David Wang,[1,†] Leon Weber-Genzel,[2] Pia Droop,[1] Benedikt Fuchs,[3] Alan Akbik[1] and Ulf Leser[1,*]

[1]Department of Computer Science, Humboldt-Universität zu Berlin, Unter den Linden 6, 10099 Berlin, Germany, [2]Center for Information and Language Processing (CIS), Ludwig Maximilian University Munich, Geschwister-Scholl-Platz 1, 80539 München, Germany and [3], Research Industrial Systems Engineering (RISE) Forschungs-, Entwicklungs- und Großprojektberatung GmbH, Concorde Business Park F, 2320 Schwechat, Austria

[*]Corresponding authors: saengema@informatik.hu-berlin.de and leser@informatik.hu-berlin.de
[†]Authors contributed equally.

FOR PUBLISHER ONLY Received on Date Month Year; revised on Date Month Year; accepted on Date Month Year

Abstract

Motivation: With the exponential growth of the life sciences literature, biomedical text mining (BTM) has become an essential technology for accelerating the extraction of insights from publications. The identification of entities in texts, such as diseases or genes, and their normalization, i.e. grounding them in knowledge base, are crucial steps in any BTM pipeline to enable information aggregation from multiple documents. However, tools for these two steps are rarely applied in the same context in which they were developed. Instead, they are applied "in the wild", i.e. on application-dependent text collections from moderately to extremely different from those used for training, varying e.g. in focus, genre or text type. This raises the question whether the reported performance, usually obtained by training and evaluating on different partitions of the same corpus, can be trusted for downstream applications.
Results: Here, we report on the results of a carefully designed *cross-corpus* benchmark for entity recognition and normalization, where tools were applied systematically to corpora not used during their training. Based on a survey of 28 published systems, we selected five, based on pre-defined criteria like feature richness and availability, for an in-depth analysis on three publicly available corpora covering four entity types. Our results present a mixed picture and show that cross-corpus performance is significantly lower than the in-corpus performance. HunFlair2, the redesigned and extended successor of the HunFlair tool, showed the best performance on average, being closely followed by PubTator Central. Our results indicate that users of BTM tools should expect a lower performance than the original published one when applying tools in "the wild" and show that further research is necessary for more robust BTM tools.
Availability and implementation: All our models are integrated into the NLP framework flair: `https://github.com/flairNLP/flair`. Code to reproduce our results is available at: `https://github.com/hu-ner/hunflair2-experiments`.
Contact: saengema@informatik.hu-berlin.de, leser@informatik.hu-berlin.de
Supplementary information: Supplementary information are available at *Bioinformatics* online.

© The Author 2022. Published by Oxford University Press. All rights reserved. For permissions, please e-mail: journals.permissions@oup.com

A. Surveyed tools

In Table 1, we present each tool found in our literature review and their exclusion criterion if not included.

A.1. Selected tools

Here we describe in details each tool selected for in-depth analysis.

BERN2 (Mujeen et al., 2022) uses a pipeline approach for entity extraction, separating NER and NEN into two distinct steps built upon each other. The NER models of BERN2 consist of a transformer-based RoBERTa model and a Conditional Random Field based on tmVar 2.0 (Wei et al., 2018). The CRF is used to extract variant entities, whereas the RoBERTa model extracts the other five entity types. The RoBERTa (Liu et al., 2019) model is trained in a multi-task fashion to share model parameters during the training of each entity type. For training the NER model, (Liu et al., 2019) use BC2GM (Smith et al., 2008) for genes, BC4CHEMD (Krallinger et al., 2015) for chemicals, NCBI Disease (Doğan et al., 2014-02) for diseases, Linnaeus (Gerner et al., 2010) for species and JNLPBA (Collier and Kim, 2004) for cell line The normalization component of BERN2 is a hybrid system that relies on a mixture of rule-based and neural-based approaches. BERN2 first tries to normalize entity mentions with rule-based models: GNormPlus (Wei et al., 2015) for genes, a sieve-based approach for diseases (D′Souza and Ng, 2015), tmChem[1] (Leaman et al.) for drug/chemical and dictionary lookup for species. If the rule-based system fails, mentions are passed to a BioSyn model (Sung et al., 2020) for genes, diseases, and chemicals. The BC2GN corpus (Smith et al., 2008) is used for training the gene normalization, BC5CDR for training chemical and disease normalization, and NCBI-Disease also for disease normalization.

bent (Ruas et al., 2023) also uses a pipeline approach separating NER and NEN into two steps. The NER component comprises standard transformer-based models initialized with weights from PubMedBERT (Gu et al., 2022). The transformers are fine-tuned separately for each entity type. Bent is trained on 15 distinct corpora for chemicals recognition[2] (BC5CDR (Li et al., 2016) and NLM-Chem(Islamaj et al., 2021b) among them), 9 corpora for disease recognition[3] (BC5CDR and NCBI-disease (Doğan et al., 2014-02) among them), 19 corpora for genes[4] (BC2GM (Smith et al., 2008) and CRAFT (Cohen et al., 2017) among them) and 9 corpora for species[5] (Linnaeus (Gerner et al., 2010) and CRAFT among them). The NEN component is based on the PageRank algorithm (Page et al., 1999). It builds a graph where nodes are names in the KB. Nodes are connected if a relation exists between them. The relations are either (i) specified in the KB (e.g., in UMLS) or (ii) automatically extracted from the training corpora. To perform mention normalization, Bent selects the node (KB name) which maximizes the coherence of the graph (Ruas et al., 2020).

PubTator is a web-based interface providing pre-computed entity annotations for all PubMed and PubMed Central documents and an API for entity extraction on custom documents. As we have no direct access to the PubTator source code and no documentation of how often updates have been applied to the public service since the last publication, we must rely on information from several publications to describe the models used for entity extraction. However, the authors regularly release new publications describing developments to the entity extraction systems so we may provide an overview of the models used according to the current publication records. We access PubTator through its API to process raw text[6], i.e., we submit plain text from the corpora to be annotated. As publications about PubTator are separated by their extracted entity type, we also report NER and NEN models for each entity type separately here. Generally, the NER and NEN systems make use of a pipelined approach separating recognition and normalization into two distinct steps. Authors of (Islamaj et al., 2021c) report that PubTator uses an updated version of GNormPlus (Wei et al., 2015), which uses the BlueBERT language model (Peng et al., 2019) for NER trained on the GnormPlus corpus (Wei et al., 2015) and NLM-Gene (Islamaj et al., 2021c). The gene normalization component is a statistical inference network (Wei and Kao, 2011) based on TF-IDF frequencies. SR4GN (Species Recognition for Gene Normalization) (Wei et al., 2012) is used for species recognition and normalization, a rule-based system which, as the name suggests, is mainly a support component for gene normalization. According to (Islamaj et al., 2021a), PubTator currently uses BlueBERT trained on BC5CDR (Li et al., 2016) and NLM-Chem (Islamaj et al., 2021a) for chemical NER, and a multi-terminology candidate resolution algorithm (MTCR) for chemical normalization, which employs multiple string-matching methods. For disease and cell lines, PubTator offers access to two TaggerOne models (Leaman and Lu, 2016) for both NER and NEN: the first trained on NCBI Disease (Doğan et al., 2014-02) and BC5CDR corpora, the second on BioID (Arighi et al., 2017).

SciSpacy uses a pipeline approach separating the NER and NEN steps. For NER, SciSpacy uses a neural network approach based on Stack LSTMs (Lample et al., 2016). It offers four distinct models trained on different biomedical corpora: BC5CDR for chemicals and diseases, CRAFT (Bada et al., 2012) for cell types, chemicals, proteins, genes, and species, JNLPBA for cell lines, cell types, DNAs, RNAs and proteins, and BioNLP13 CG (Pyysalo et al., 2013) for chemicals, diseases, genes, and species. The normalization component included in the tool leverages a string-matching approach based on characters 3-grams. SciSpacy also uses a dedicated abbreviation resolution module, which identifies and expands abbreviations in the text to increase further downstream normalization performance. SciSpacy allows linking to multiple KBs. We use UMLS (Bodenreider, 2004) since it covers all entity types and KBs used in our cross-corpus

[1] without abbreviation resolution by Ab3P (Sohn et al., 2008)

[2] https://huggingface.co/pruas/BENT-PubMedBERT-NER-Chemical

[3] https://huggingface.co/pruas/BENT-PubMedBERT-NER-Disease

[4] https://huggingface.co/pruas/BENT-PubMedBERT-NER-Gene

[5] https://huggingface.co/pruas/BENT-PubMedBERT-NER-Organism

[6] https://www.ncbi.nlm.nih.gov/research/pubtator/api.html - accessed on the 2023/07/21

Table 1. List of entity extraction tools identified by our literature review. For the non-selected tools we report the unfullfilled criteria (see 2.1) disqualifying from our evaluation. Abbreviations used in the table are: "RB" for rule-based systems, "ML" denote all standard machine learning approaches (e.g. Support Vector Machines) distinguished from neural-network based ("N") ones. Entity types we consider are: genes (Ge), species (Sp), disease (Di), chemical (Ch), cell line (Cl), variant (Va). We also include a miscellaneous category (Misc.) if the tools supports additional (clinical) entity types. ✓ denotes support for both NER and NEN, (✓) denotes support for either NER or NEN only. Last update refers to the date when the corresponding GitHub repository was changed last. Citation count refers to the number of Google scholar citations as of the 10/01/2024. † Requires UMLS, which has its own license ‡ Commercial

Tools	Ref.	API	Ge	Sp	Di	Ch	Cl	Va	Misc.	NER	NEN	Pub. year	Last update	Citations	License	Exclusion
**Non-selected**																C1, C3
BANNER	Leaman and Gonzalez (2007)	Java	(✓)		(✓)					ML	-	2007	-	635	CPL	C1
EasyNER	Ahmed et al. (2023)	Python	(✓)	(✓)	(✓)	(✓)	(✓)			N	-	2023	12/2023	1	Apache-2.0 license	C1, C2, C3
Gimli	Campos et al. (2013b)	Java	(✓)				(✓)			RB + ML	-	2013	-	136	-	C1, C3
Med7	Kormilitzin et al. (2021)	Python							(✓)	N + ML	-	2021	12/2021	86	Apache 2.0	C1, C2, C3
MedTagger	Wen et al. (2019)	Java							(✓)	RB	-	2019	10/2023	77	Apache-2.0 license	C1, C3
NeuroNER	Dernoncourt et al. (2017)	Python							(✓)	N	-	2017	10/2019	241	MIT license	C1
SparkNLP	Kocaman and Talby (2021)	Python,R,Scala,Java	(✓)	(✓)	(✓)	(✓)	(✓)		(✓)	N	-	2021	01/2024	54	Apache 2.0 ‡	C1
Stanza	Zhang et al. (2021)	Python	(✓)	(✓)	(✓)	(✓)	(✓)		(✓)	N	-	2021	12/2023	118	Apache 2.0	C1, C3
TNER	Ushio and Camacho-Collados (2021)	Python	(✓)				(✓)			N	-	2021	05/2023	51	MIT license	C1, C3
ChatGPT	Ouyang et al. (2022)	Web/REST								N	-	2022	-	3952	ToS	C1, C3
Gilda	Gyori et al. (2022)	Python/REST	(✓)						(✓)	-	RB + ML	2022	12/2023	8	BSD2	C2
Bio-YODIE	Gorrell et al. (2018)	Java		✓	✓	✓				RB + N	RB + N	2018	10/2019	22	GNU Affero GPL	C2
CLAMP	Soysal et al. (2018)	Java	✓	✓	✓	✓	✓	✓	✓	ML + RB	RB	2018	-	358	Free for research use	C2, C3, C4
cTAKES	Savova et al. (2010)	Java	✓	✓	✓		✓	✓	✓	RB	RB	2010	-	2148	Apache License V2.0 †	C2, C3, C4
HITEx	Zeng et al. (2006)	Java							✓	ML + RB	RB	2006	-	433	Open source, i2b2 †	C2
MedCat	Kraljevic et al. (2021)	Python		✓	✓	✓			✓	RB	RB + N	2021	12/2023	107	Elastic License 2.0	C2
MedSpaCy	Eyre et al. (2021)	Python							✓	N + ML + RB	RB	2021	01/2024	51	MIT license	C2, C3
MetaMap Lite	Demner-Fushman et al. (2017)	Java/Web+REST	✓	✓	✓	✓	✓	✓	✓	RB	RB	2015	07/2022	172	BSD license †	C2, , C4
Neji	Campos et al. (2013a)	Java	✓	✓	✓	✓	✓			RB + ML	RB	2013	05/2017	93	CC BY-NC-SA 3.0	C2
OnTheFly2.0	Baltoumas et al. (2021)	Web	✓	✓	✓				✓	RB	RB	2021	07/2022	14	GPLv3	C2, C3
QuickUMLS	Soldaini and Goharian (2016)	Python	✓	✓	✓	✓	✓	✓	✓	RB	RB	2016	10/2023	215	MIT license †	C2, C4
Bio-NLP	Badenes-Olmedo et al. (2022)	Python, Web	✓		✓	✓				N	RB	2022	03/2022	2	Apache-2.0 license	C3
**Selected**																
PubTator Central	Wei et al. (2019)	REST/Tools	✓	✓	✓	✓	✓	✓		ML / N	RB	2019	-	315	N/A	
BERN2	Mujeen et al. (2022); Kim et al. (2019)	Python/Web	✓	✓	✓	✓	✓	✓	✓	N	RB + N	2022	11/2023	46	BSD 2-Clause "Simplified"	
SciSpacy	Neumann et al. (2019)	Python	(✓)	✓	✓	✓	✓	(✓)	✓	N	RB	2019	10/2023	635	Apache 2.0	
bent	Ruas et al. (2020, 2023)	Python	✓	✓	✓	✓	✓	(✓)	✓	N	RB	2020	12/2023	13	Apache 2.0	
HunFlair2		Python	✓	✓	✓	✓	✓			N	RB + N	2021	01/2024	83	MIT License	

experiments (see Section 2). We note, however, that due to the UMLS licensing, SciSpacy comes only with a subset of the resources of UMLS, namely those categorized as in levels 0, 1, 2, and 9 from the license[7].

B. Corpora

BioID was created for the BioCreative VI shared task "Track 1: Interactive Bio-ID Assignment (IAT-ID)". Participants of the task were ask to annotate text originating from figure captions with the entity types and IDs for organisms, genes, proteins, miRNA, small molecules, cellular components, cell types and cell lines, tissues and organs. The corpus contains annotated captions for a total of 570 articles. For NER, in our evaluation, we make use of the chemical, gene and organism (species) annotations given in the data. Note that we do not use the cell line annotations, because one of the selected tools (PubTator) uses these annotations during training and testing. For entity extraction use only the species entity type, as other entities are normalized to KBs not supported by the tools selected.

tmVar v3 contains 500 abstracts of PubMed articles manually annotated with different types of genetic variant and gene mentions. In total, the corpus contains over 4,000 mentions of genes as well as their NCBI gene identifiers. In our study, we use the gene entity mentions both evaluations.

MedMentions consists of 4,392 PubMed abstracts, which were randomly chosen from the papers published on PubMed in 2016. The corpus provides annotations of entity mentions linked to UMLS spanning all its Semantic Types (entity types). We use the the ST21pv (21 Semantic Types and Preferred Vocabularies) split. For both NER and NEN, we evaluate on chemical and disease entities which we can be mapped to CTD identifiers via UMLS cross-reference tables.

C. Macro F1-score calculation

In practice, our macro F1-score calculation looks a bit different then presented in Section 4.2. Instead of calculating individual F1-scores for every database entity, we first compute individual precision and recall scores for each entity whenever possible. We then average those scores to obtain macro precision and macro recall and only then calculate their harmonic mean as our macro F1-score. This adapted calculation has a simple rationale: For rarely occurring entities in a given corpus, e.g., zebrafish ("NCBI taxon 7955"), there might often either be no recall or no precision score defined. In our zebrafish example, there might be a false positive prediction for zebrafish in a given corpus but neither true positives nor false negatives as the zebrafish entity might have never been part of the original corpus. This means there is no individual recall score to be defined for the zebrafish entity and thus no F1-score. However, we can still take into account the precision score defined for this entity (0 in this case) into the calculation of the macro precision score as a whole.

D. Comparison of HunFlair to HunFlair2

Table 2. Comparing F1-scores for NER in HunFlair versions 1 and 2.

**Entity type / dataset**	**HunFlair2**	**HunFlair-v1**	**Difference**
Chemical			
MedMentions	65.70	58.13	*+7.57*
Disease			
MedMentions	66.24	66.79	*-0.55*
Gene			
tmVar (v3)	87.87	83.21	*+4.66*
Species			
BioID	58.21	57.62	*+0.59*
Avg. All	69.51	66.44	*+3.07*

In Table 2, we report the comparison of results between HunFlair and HunFlair2. The disease entities are the only ones who do not profit from a joint NER model as they lose on average -1.57 pp on the F1 score compared to the disease-specific entity recognizer in HunFlair. Overall, HunFlair2 achieves a 2.02 pp improvement over HunFlair averaged over all entity types.

E. Named entity recognition

E.1. Named entity recognition: Evaluation

Following previous studies Giorgi and Bader (2020); Weber et al. (2021) we report F1 scores comparing predicted spans to gold ones. We classify a predicted span as a true positive (TP) if it either (i) matches exactly a gold span or (ii) differs with the gold span by only one

[7] https://uts.nlm.nih.gov/uts/license/license-category-help.html

Table 3. Best SciSpacy NER model on each of our evaluation corpora.

Dataset	SciSpacy model
MedMentions (Chemical)	en_ner_bc5cdr_md
MedMentions (Disease)	en_ner_bc5cdr_md
tmVar (v3)	en_ner_bionlp13cg_md
BioID (Species)	en_ner_craft_md

character either at the beginning or the end. This is to account different handling of special characters by different tools which may result in minor span differences. To ensure maximal fairness, we have identified and removed all sentences (and documents) in the corpora used to train HunFlair2 which present an overlap with the corpora selected for our benchmarking. Overlap is computed by matching all strings in each corpora on a sentence level. We note that the only significant overlap we found was between BioRED and tmVar v3 with 2,172 out of 6,755 sentences overlapping. For the evaluations of the SciSpacy on our corpora, we compute results using all its available NER models[8] (en_ner_bc5cdr_md, en_ner_bionlp13cg_md, en_ner_craft_md, en_ner_jnlpba_md) and report the result of the one that performs the best on the given corpora. Table 3 reports the combinations of SciScpacy model and corpus.

E.2. Named entity recognition: Results

Table 4. Mention-level named entity recognition (NER) results evaluated by F1 scores. For each tool we report the performance differences between NER and the end-to-end entity normalization results in parenthesis. Bold figures highlight the highest value per row.

	BERN2	HunFlair2	PubTator	SciSpacy	bent
Chemical					
MedMentions	56.07	**65.70**	43.07	49.65	57.78
	(+12.70)	*(+11.59)*	*(+11.02)*	*(+13.33)*	*(+15.21)*
Disease					
MedMentions	62.95	**66.24**	50.08	53.62	65.22
	(+14.41)	*(+6.87)*	*(+8.05)*	*(+11.77)*	*(+18.02)*
Gene					
tmVar (v3)	48.05	87.87	**88.82**	65.26	82.27
	(+4.09)	*(+11.12)*	*(+2.80)*	-	*(+81.73)*
Species					
BioID	60.80	58.21	60.35	43.94	**64.91**
	(+46.45)	*(+8.55)*	*(+1.45)*	*(+6.80)*	*(+54.56)*
Avg. All	56.97	**69.51**	60.58	53.12	67.55
	(+20.11)	*(+9.53)*	*(+5.83)*	*(+14.68)*	*(+42.38)*

In Table 4 we report Table 5 the NER results by corpus for all the tools with standard (2.3) and lenient (4.3) evaluation, respectively.

E.3. Macro-average named entity recognition

Similarly to the infrequent entity analysis conducted for joint entity extraction in Section 4.2, we conducted an analysis for infrequent entities for NER. The results can be found in Table 6. We observe a consistent drop in F1-score performance of about 26 pp compared to the micro F1-score evaluation in Table 4 across all tools. This shows that infrequent entities are not recognized as well as frequent ones indicating a need to further improve the capabilities of current biomedical NER models to handle those low-tail entities.

F. Named entity extraction results

F.1. Micro-average named entity extraction

In Table 7 we report the detailed cross-corpus micro-average precision, recall and F1 score of all evaluated tools.

F.2. Macro-average named entity extraction

In Table 8 we report the macro-average F1 scores of all evaluated tools in the full named entity extraction setting.

Table 5. Named entity recognition results using a lenient evaluation setting, i.e. we count each prediction as true positive which is a sub- or superstring of gold standard entity mention. For each tool we report the performance differences between the strict and lenient NER results in parenthesis.

	**BERN2**	**HunFlair2**	**PubTator**	**SciSpacy**	**bent**
Chemical					
MedMentions	65.37	**71.77**	52.69	57.23	66.60
	(+9.30)	*(+6.07)*	*(+9.62)*	*(+7.58)*	*(+8.82)*
Disease					
MedMentions	72.03	**75.30**	64.17	66.78	74.71
	(+9.08)	*(+9.06)*	*(+14.09)*	*(+13.16)*	*(+9.49)*
Gene					
tmVar (v3)	88.76	**92.35**	89.46	74.44	89.48
	(+40.71)	*(+4.48)*	*(+0.64)*	*(+9.18)*	*(+7.21)*
Species					
BioID	**68.46**	64.99	62.31	48.39	67.82
	(+7.66)	*(+6.78)*	*(+1.96)*	*(+4.45)*	*(+2.91)*
Avg. All	73.66	**76.10**	67.16	61.71	74.65
	(+16.69)	*(+6.60)*	*(+6.58)*	*(+8.59)*	*(+7.11)*

Table 6. Macro F1 scores for Named Entity Recognition over unique mentions. We compare the results to the micro-average F1 scores for NER in Table 4.

	**BERN2**	**HunFlair2**	**PubTator**	**SciSpacy**	**bent**
Chemical					
MedMentions	39.05	47.36	26.90	30.55	41.58
	(-17.02)	*(-18.34)*	*(-16.17)*	*(-19.10)*	*(-16.20)*
Disease					
MedMentions	37.03	39.82	23.97	26.82	37.52
	(-25.92)	*(-26.42)*	*(-26.11)*	*(-26.80)*	*(-27.70)*
Gene					
tmVar (v3)	26.88	66.74	72.01	38.42	58.42
	(-21.17)	*(-21.13)*	*(-16.81)*	*(-26.84)*	*(-23.85)*
Species					
BioID	14.67	15.39	16.67	6.51	25.78
	(-46.13)	*(-42.82)*	*(-43.68)*	*(-37.43)*	*(-39.13)*
Avg. All	29.41	42.33	34.89	25.58	40.83
	(-27.56)	*(-27.18)*	*(-25.69)*	*(-27.54)*	*(-26.72)*

F.3. Document-level name entity extraction

For a prediction to be correct the evaluation applied in Section 3 requires a match between both the mention boundaries and the normalization identifier with the gold standard. This is a significantly challenging scenario, especially for a cross-corpus setting, where different annotation guidelines impact what constitutes a mention. To account for a more lenient type of evaluation, we report *document-level* performance of all tools in Table 9. That is, we compare the set of unique gold standard and predicted identifiers gathered from all mentions of a document. This type of evaluation also matches the use case of semantic indexing, a common application of entity extraction, in which all concepts mentioned in a document are indexed for improved information retrieval Leaman et al. (2023).

The results of the mention-level and document-level evaluation are not directly comparable, but it is shown that the tools can essentially achieve higher results. One would expect that the document-level results would have to increase more for longer texts, since in these multiple mentions of the same entities occur likely more frequently and in a document-oriented evaluation the incorrect or missing identification of individual mentions is not taken into account. Moreover, the recognition of exact mention boundaries doesn't impact the metric. The considerations are confirmed by our results. For instance, we see strong improvements for extraction of disease across all five tools. The only exception is the recognition of species. In this case, better results can only be achieved for two of the five tools (BERN2 and bent).

[8] See https://allenai.github.io/scispacy/ for details.

Table 7. Cross-corpus micro-average precision, recall and F1 score of all tools evaluated tools.

	BERN2			PTC			SciSpacy			bent			HunFlair2		
	**P**	**R**	**F1**	**P**	**R**	**F1**	**P**	**R**	**F1**	**P**	**R**	**F1**	**P**	**R**	**F1**
Chemical															
MedMentions	52.48	36.96	43.37	58.84	50.08	54.11	42.38	25.77	32.05	45.45	30.25	36.32	51.36	36.36	42.57
Disease															
MedMentions	45.13	52.51	48.54	53.66	66.44	59.37	42.15	41.92	42.03	40.33	43.48	41.85	43.34	51.81	47.20
Gene															
tmvar (v3)	40.37	48.26	43.96	74.58	79.06	76.75	90.33	82.11	86.02	-	-	-	0.52	0.57	0.54
Species															
BioID	17.93	11.96	14.35	65.94	39.83	49.66	81.52	46.11	58.90	50.66	29.31	37.14	11.79	9.22	10.35
Avg. All	38.98	37.42	37.56	63.25	58.85	59.97	64.09	48.98	54.75	45.48	34.35	38.43	26.75	24.49	25.16

Table 8. Macro F1 scores for named entity extraction, i.e., end-to-end entity recognition and normalization. We compute precision and recall for each entity in the KB and take the harmonic mean (F1). We compare the results to micro F1 scores in Table 3 (difference in brackets).

	**BERN2**	**HunFlair2**	**PubTator**	**SciSpacy**	**bent**
Chemical					
MedMentions	32.33	**36.82**	22.81	32.66	30.16
	(-11.04)	*(-17.29)*	*(-9.24)*	*(-3.66)*	*(-12.41)*
Disease					
MedMentions	31.51	**39.94**	26.47	36.93	24.84
	(-17.03)	*(-19.43)*	*(-15.56)*	*(-4.92)*	*(-22.36)*
Gene					
tmVar (v3)	25.51	47.47	**72.46**	-	0.31
	(-18.45)	*(-29.28)*	*(-13.56)*	(-)	*(-0.23)*
Species					
BioID	16.83	8.38	**20.73**	4.78	3.25
	(+2.48)	*(-41.28)*	*(-38.17)*	*(-32.36)*	*(-7.10)*
Avg	26.54	33.15	35.62	24.79	14.64
	(-11.01)	*(-26.82)*	*(-19.13)*	*(-13.65)*	*(-10.52)*

Table 9. Cross-corpus evaluation results for *document-level* named entity extraction. Results are micro-F1 (average over entities) score on the entire corpora. In parenthesis we report the difference with the *mention-level* performance.

	**BERN2**	**HunFlair2**	**PTC**	**SciSpacy**	**bent**
Chemical					
MedMentions	41.86	**48.93**	35.20	37.90	41.90
	(-1.51)	*(-5.18)*	*(3.15)*	*(1.58)*	*(-0.67)*
Disease					
MedMentions	51.35	**64.58**	56.72	55.86	55.57
	(+2.81)	*(+5.21)*	*(+14.69)*	*(+14.01)*	*(+8.37)*
Gene					
tmVar v3	72.52	79.03	**88.58**	-	0.64
	(+28.56)	*(+2.28)*	*(+2.56)*	-	*(+0.10)*
Species					
BioID	16.38	49.49	**54.50**	36.72	12.11
	(+2.03)	*(-2.17)*	*(-4.40)*	*(-0.42)*	*(+1.76)*
Avg	45.53	60.00	58.75	43.49	27.56
	(+7.97)	*(+0.03)*	*(+4.00)*	*(+5.06)*	*(+2.40)*

G. Mapping from UMLS to CTD chemicals/diseases

Table 10. Overview of the top-50 disease and chemical UMLS concepts that can't be mapped to MESH.

Chemicals			Disease		
**Concept Identifier**	**Name**	**Frequency**	**Concept Identifier**	**Name**	**Frequency**
C1254351	Pharmacologic Substance	783	C0243095	Finding	1925
C0033684	Proteins	693	C0011900	Diagnosis	535
C0014442	Enzymes	282	C0150312	Present	352
C0013227	Pharmaceutical Preparations	257	C1457887	Symptoms	328
C1101610	MicroRNAs	257	C0442726	Detected (finding)	321
C0870883	Metabolite	190	C0184511	Improved	317
C0035696	RNA, Messenger	182	C0035648	risk factors	290
C0012854	DNA	174	C1446409	Positive	269
C0005479	Biomaterials	169	C0205160	Negative	212
C1764827	Isolate - microorganism	169	C0221198	Lesion	206
C0574031	Biologically active substance	151	C0332128	Examined	186
C0003241	Antibodies	151	C1513916	Negative Finding	178
C0032521	Polymers	132	C0033213	Problem	169
C1519595	Transcript	94	C0600688	Toxic effect	139
C0597177	Particle	90	C0449416	Source	138
C0040648	TRANSCRIPTION FACTOR	88	C0456984	Test Result	126
C1706082	Compound (substance)	82	C4055506	Accumulation	119
C0243077	inhibitors	82	C0439663	Infected	119
C0010454	Culture Media	79	C0449381	Observation parameter	116
C0035668	RNA	78	C0038435	Stress	105
C1696465	placebo	73	C1306577	Death (finding)	102
C0597357	receptor	73	C3714660	Trauma	100
C0220806	Chemicals	71	C0332167	High risk of	97
C0079904	NF-kappa B	66	C1858460	Maternal	82
C0032136	Plasmids	62	C1273937	Not significant	76
C0025938	Micelles	61	C1444656	Indicated	74
C0013161	Drug Carriers	61	C0003467	Anxiety	69
C0444626	Crystal Structure	61	C1514241	Positive Finding	67
C1704241	complex (molecular entity)	58	C0332461	Plaque (lesion)	67
C0023688	Ligands	58	C0178314	Poisoning / injury	66
C0086860	promoter	56	C0205161	Abnormal	65
C1099354	RNA, Small Interfering	56	C0750484	Confirmation	62
C0596235	Calcium ion	53	C0086132	Depressive Symptoms	53
C0009325	Collagen	52	C1269955	Tumor Cell Invasion	52
C0020852	Immunoglobulin G	51	C0221423	Illness (finding)	51
C0289507	NF-E2-Related Factor 2	49	C1457868	Worse	50
C3539881	gamma-interferon	48	C0332149	Possible	49
C0034861	Recombinant Proteins	48	C3263722	Non-/Traumatic injury	48
C0023764	Lipase	46	C0007465	Cause of Death	47
C0004358	Autoantibodies	44	C1704258	Abnormality	47
C0003250	Monoclonal Antibodies	44	C0231170	Disability	45
C0074529	Silk	43	C0231221	Asymptomatic	43
C0181074	Graft material	42	C0262926	Medical History	41
C0600388	Extracellular Signal Reg. Kinases	41	C0442797	Decreasing	40
C0013162	Drug Combinations	40	C0015663	Fasting	40
C1510464	Protein Structure	38	C0087130	Uncertainty	37
C0011379	Dental Materials	37	C2825142	Experimental Result	33
C0887909	RNA, Untranslated	37	C0241863	Diabetic	33
C0164786	Proto-Oncogene Proteins c-akt	37	C3714625	Neuropathic pain	33
C0253050	Stat3 protein	36	C0442739	No status change	32

References

R. Ahmed, P. Berntsson, A. Skafte, S. K. Rashed, M. Klang, A. Barvesten, O. Olde, W. Lindholm, A. L. Arrizabalaga, P. Nugues, et al. Easyner: A customizable easy-to-use pipeline for deep learning-and dictionary-based named entity recognition from medical text. *arXiv preprint arXiv:2304.07805*, 2023.

C. Arighi, L. Hirschman, T. Lemberger, S. Bayer, R. Liechti, D. Comeau, and C. Wu. Bio-id track overview. In *BioCreative VI Challenge Evaluation Workshop*, volume 482, page 376, 2017.

M. Bada, M. Eckert, D. Evans, K. Garcia, K. Shipley, D. Sitnikov, W. A. Baumgartner, K. B. Cohen, K. Verspoor, J. A. Blake, et al. Concept annotation in the craft corpus. *BMC bioinformatics*, 13(1):1–20, 2012.

C. Badenes-Olmedo, Á. Alonso, and O. Corcho. An overview of drugs, diseases, genes and proteins in the cord-19 corpus. *Procesamiento del Lenguaje Natural*, 69:165–176, 2022.

F. A. Baltoumas, S. Zafeiropoulou, E. Karatzas, S. Paragkamian, F. Thanati, I. Iliopoulos, A. G. Eliopoulos, R. Schneider, L. J. Jensen, E. Pafilis, and G. A. Pavlopoulos. Onthefly2.0: a text-mining web application for automated biomedical entity recognition, document annotation, network and functional enrichment analysis. *NAR Genomics and Bioinformatics*, 3, 10 2021. doi: 10.1093/nargab/lqab090. URL http://dx.doi.org/10.1093/nargab/lqab090.

O. Bodenreider. The unified medical language system (umls): integrating biomedical terminology. *Nucleic Acids Research*, 32:267D–270, 1 2004. URL http://dx.doi.org/10.1093/nar/gkh061.

D. Campos, S. Matos, and J. L. Oliveira. A modular framework for biomedical concept recognition. *BMC Bioinformatics*, 14, 2013a. URL http://dx.doi.org/10.1186/1471-2105-14-281.

D. Campos, S. Matos, and J. L. Oliveira. Gimli: open source and high-performance biomedical name recognition. *BMC Bioinformatics*, 14(1):54, 2013b. URL https://doi.org/10.1186/1471-2105-14-54.

K. B. Cohen, K. Verspoor, K. Fort, C. Funk, M. Bada, M. Palmer, and L. E. Hunter. The colorado richly annotated full text (craft) corpus: Multi-model annotation in the biomedical domain. *Handbook of Linguistic annotation*, pages 1379–1394, 2017.

N. Collier and J.-D. Kim. Introduction to the bio-entity recognition task at jnlpba. In *Int. Joint Workshop on Natural Language Processing in Biomedicine and its Applications*, pages 73–78, 2004.

D. Demner-Fushman, W. J. Rogers, and A. R. Aronson. MetaMap lite: an evaluation of a new java implementation of MetaMap. *Journal of the American Medical Informatics Association*, 24(4):841–844, 2017. URL https://academic.oup.com/jamia/article/24/4/841/2961848.

J. D′Souza and V. Ng. Sieve-based entity linking for the biomedical domain. In *Proceedings of the 53rd Annual Meeting of the Association for Computational Linguistics and the 7th International Joint Conference on Natural Language Processing (Volume 2: Short Papers)*, volume 9, pages 297–302, Beijing, China, 2015. Association for Computational Linguistics. URL https://aclanthology.org/P15-2049.

F. Dernoncourt, J. Y. Lee, and P. Szolovits. Neuroner: an easy-to-use program for named-entity recognition based on neural networks. In *Proceedings of the 2017 Conference on Empirical Methods in Natural Language Processing: System Demonstrations*, pages 97–102, 2017.

R. I. Doğan, R. Leaman, and Z. Lu. Ncbi disease corpus: A resource for disease name recognition and concept normalization. *Journal of Biomedical Informatics*, 47:1–10, 2014-02. URL https://www.sciencedirect.com/science/article/pii/S1532046413001974.

H. Eyre, A. B. Chapman, K. S. Peterson, J. Shi, P. R. Alba, M. M. Jones, T. L. Box, S. L. DuVall, and O. V. Patterson. Launching into clinical space with medspacy: a new clinical text processing toolkit in python. In *AMIA Annual Symposium Proceedings*, volume 2021, page 438. American Medical Informatics Association, 2021.

M. Gerner, G. Nenadic, and C. M. Bergman. Linnaeus: a species name identification system for biomedical literature. *BMC bioinformatics*, 11(1):1–17, 2010.

J. M. Giorgi and G. D. Bader. Towards reliable named entity recognition in the biomedical domain. *Bioinformatics*, 36(1):280–286, 2020.

G. Gorrell, X. Song, and A. Roberts. Bio-yodie: A named entity linking system for biomedical text. *CoRR*, abs/1811.04860, 2018. URL http://arxiv.org/abs/1811.04860.

Y. Gu, R. Tinn, H. Cheng, M. Lucas, N. Usuyama, X. Liu, T. Naumann, J. Gao, and H. Poon. Domain-specific language model pretraining for biomedical natural language processing. *ACM Trans. Comput. Heal.*, 3(1):2:1–2:23, 2022. URL https://doi.org/10.1145/3458754.

B. M. Gyori, C. T. Hoyt, and A. Steppi. Gilda: biomedical entity text normalization with machine-learned disambiguation as a service. *Bioinformatics Advances*, 2(1):vbac034, 2022. URL https://doi.org/10.1093/bioadv/vbac034.

R. Islamaj, R. Leaman, S. Kim, D. Kwon, C. Wei, D. Comeau, Y. Peng, D. Cissel, C. Coss, C. Fisher, et al. Nlm-chem, a new resource for chemical entity recognition in pubmed full text literature. *Scientific Data*, 8(1):91–91, 2021a.

R. Islamaj, R. Leaman, S. Kim, D. Kwon, C.-H. Wei, D. C. Comeau, Y. Peng, D. Cissel, C. Coss, C. Fisher, et al. Nlm-chem, a new resource for chemical entity recognition in pubmed full text literature. *Scientific data*, 8(1):91, 2021b.

R. Islamaj, C.-H. Wei, D. Cissel, N. Miliaras, O. Printseva, O. Rodionov, K. Sekiya, J. Ward, and Z. Lu. Nlm-gene, a richly annotated gold standard dataset for gene entities that addresses ambiguity and multi-species gene recognition. *Journal of biomedical informatics*, 118:103779, 2021c. URL https://doi.org/10.1016/j.jbi.2021.103779.

D. Kim, J. Lee, C. H. So, H. Jeon, M. Jeong, Y. Choi, W. Yoon, M. Sung, and J. Kang. A neural named entity recognition and multi-type normalization tool for biomedical text mining. *IEEE Access*, 7:73729–73740, 2019.

V. Kocaman and D. Talby. Biomedical named entity recognition at scale. In *International Conference on Pattern Recognition*, pages 635–646, 2021.

A. Kormilitzin, N. Vaci, Q. Liu, and A. Nevado-Holgado. Med7: A transferable clinical natural language processing model for electronic health records. *Artificial Intelligence in Medicine*, 118:102086, 2021.

Z. Kraljevic, T. Searle, A. Shek, L. Roguski, K. Noor, D. Bean, A. Mascio, L. Zhu, A. A. Folarin, A. Roberts, R. Bendayan, M. P. Richardson, R. Stewart, A. D. Shah, W. K. Wong, Z. Ibrahim, J. T. Teo, and R. J. Dobson. Multi-domain clinical natural language processing with medcat: The medical concept annotation toolkit. *Artificial Intelligence in Medicine*, 117:102083, 2021. URL http://dx.doi.org/10.1016/j.artmed.2021.102083.

M. Krallinger, O. Rabal, F. Leitner, M. Vazquez, D. Salgado, Z. Lu, R. Leaman, Y. Lu, D. Ji, D. M. Lowe, et al. The chemdner corpus of chemicals and drugs and its annotation principles. *Journal of cheminformatics*, 7(1):1–17, 2015.

G. Lample, M. Ballesteros, S. Subramanian, K. Kawakami, and C. Dyer. Neural architectures for named entity recognition. In *Proceedings of the 2016 Conference of the North American Chapter of the Association for Computational Linguistics: Human Language Technologies*, pages 260–270, 2016.

R. Leaman and G. Gonzalez. BANNER: AN EXECUTABLE SURVEY OF ADVANCES IN BIOMEDICAL NAMED ENTITY RECOGNITION. In *Biocomputing 2008*, pages 652–663. WORLD SCIENTIFIC, 2007. ISBN 978-981-277-608-2 978-981-277-613-6. URL http://www.worldscientific.com/doi/abs/10.1142/9789812776136_0062.

R. Leaman and Z. Lu. Taggerone: joint named entity recognition and normalization with semi-markov models. *Bioinformatics*, 32(18): 2839–2846, 2016. URL https://doi.org/10.1093/bioinformatics/btw343.

R. Leaman, C.-H. Wei, and Z. Lu. tmchem: a high performance approach for chemical named entity recognition and normalization. 7 (S1):S3.

R. Leaman, R. Islamaj, V. Adams, M. A. Alliheedi, J. a. R. Almeida, R. Antunes, R. Bevan, Y.-C. Chang, A. Erdengasileng, M. Hodgskiss, R. Ida, H. Kim, K. Li, R. E. Mercer, L. Mertová, G. Mobasher, H.-C. Shin, M. Sung, T. Tsujimura, W.-C. Yeh, and Z. Lu. Chemical identification and indexing in full-text articles: an overview of the nlm-chem track at biocreative vii. *Database*, 2023, 2023. URL http://dx.doi.org/10.1093/database/baad005.

J. Li, Y. Sun, R. J. Johnson, D. Sciaky, C.-H. Wei, R. Leaman, A. P. Davis, C. J. Mattingly, T. C. Wiegers, and Z. Lu. Biocreative v cdr task corpus: a resource for chemical disease relation extraction. *Database*, 2016(baw068), 2016. URL https://doi.org/10.1093/database/baw068.

Y. Liu, M. Ott, N. Goyal, J. Du, M. Joshi, D. Chen, O. Levy, M. Lewis, L. Zettlemoyer, and V. Stoyanov. Roberta: A robustly optimized bert pretraining approach. *arXiv preprint arXiv:1907.11692*, 2019.

S. Mujeen, J. Minbyul, C. Yonghwa, K. Donghyeon, L. Jinhyuk, and K. Jaewoo. Bern2: an advanced neural biomedical named entity recognition and normalization tool. *Bioinformatics*, 38, 2022. URL https://academic.oup.com/bioinformatics/article/38/20/4837/6687126.

M. Neumann, D. King, I. Beltagy, and W. Ammar. Scispacy: fast and robust models for biomedical natural language processing. *arXiv preprint arXiv:1902.07669*, 2019.

L. Ouyang, J. Wu, X. Jiang, D. Almeida, C. L. Wainwright, P. Mishkin, C. Zhang, S. Agarwal, K. Slama, A. Ray, J. Schulman, J. Hilton, F. Kelton, L. Miller, M. Simens, A. Askell, P. Welinder, P. F. Christiano, J. Leike, and R. Lowe. Training language models to follow instructions with human feedback. In *NeurIPS*, 2022. URL http://papers.nips.cc/paper_files/paper/2022/hash/b1efde53be364a73914f58805a001731-Abstract-Conference.html.

L. Page, S. Brin, R. Motwani, and T. Winograd. The pagerank citation ranking : Bringing order to the web. In *The Web Conference*, 1999. URL https://api.semanticscholar.org/CorpusID:1508503.

Y. Peng, S. Yan, and Z. Lu. Transfer learning in biomedical natural language processing: An evaluation of bert and elmo on ten benchmarking datasets. In *Proceedings of the 18th BioNLP Workshop and Shared Task*, pages 58–65, Florence, Italy, 2019. Association for Computational Linguistics. URL https://aclanthology.org/W19-5006.

S. Pyysalo, T. Ohta, and S. Ananiadou. Overview of the cancer genetics (cg) task of bionlp shared task 2013. In *Proceedings of the BioNLP Shared Task 2013 Workshop*, pages 58–66, 2013.

P. Ruas, A. Lamurias, and F. M. Couto. Linking chemical and disease entities to ontologies by integrating pagerank with extracted relations from literature. *Journal of Cheminformatics*, 12, 2020. URL http://dx.doi.org/10.1186/s13321-020-00461-4.

P. Ruas, D. F. Sousa, A. Neves, C. Cruz, and F. M. Couto. Lasige and unicage solution to the nasa litcoin nlp competition. *arXiv:2308.05609*, 2023.

G. K. Savova, J. J. Masanz, P. V. Ogren, J. Zheng, S. Sohn, K. C. Kipper-Schuler, and C. G. Chute. Mayo clinical text analysis and knowledge extraction system (ctakes): architecture, component evaluation and applications. *Journal of the American Medical Informatics Association*, 17:507–513, 9 2010. doi: 10.1136/jamia.2009.001560. URL http://dx.doi.org/10.1136/jamia.2009.001560.

L. Smith, L. K. Tanabe, C.-J. Kuo, I. Chung, C.-N. Hsu, Y.-S. Lin, R. Klinger, C. M. Friedrich, K. Ganchev, M. Torii, et al. Overview of biocreative ii gene mention recognition. *Genome biology*, 9(2):1–19, 2008.

S. Sohn, D. C. Comeau, W. Kim, and W. J. Wilbur. Abbreviation definition identification based on automatic precision estimates. *BMC Bioinformatics*, 9, 12 2008. doi: 10.1186/1471-2105-9-402. URL http://dx.doi.org/10.1186/1471-2105-9-402.

L. Soldaini and N. Goharian. Quickumls: a fast, unsupervised approach for medical concept extraction. In *MedIR workshop, sigir*, pages 1–4, 2016.

E. Soysal, J. Wang, M. Jiang, Y. Wu, S. Pakhomov, H. Liu, and H. Xu. CLAMP – a toolkit for efficiently building customized clinical natural language processing pipelines. *Journal of the American Medical Informatics Association*, 25(3):331–336, 2018. URL https://academic.oup.com/jamia/article/25/3/331/4657212.

M. Sung, H. Jeon, J. Lee, and J. Kang. Biomedical entity representations with synonym marginalization. In *Annual Meeting of the Association for Computational Linguistics*, page 3641–3650, 2020. URL https://aclanthology.org/2020.acl-main.335.

A. Ushio and J. Camacho-Collados. T-NER: An all-round python library for transformer-based named entity recognition. In *Proceedings of the 16th Conference of the European Chapter of the Association for Computational Linguistics: System Demonstrations*, pages 53–62, 2021. URL http://arxiv.org/abs/2209.12616.

L. Weber, M. Sänger, J. Münchmeyer, M. Habibi, U. Leser, and A. Akbik. Hunflair: an easy-to-use tool for state-of-the-art biomedical named entity recognition. *Bioinformatics*, 37(17):2792–2794, 2021.

C.-H. Wei and H.-Y. Kao. Cross-species gene normalization by species inference. *BMC Bioinformatics*, 12, 2011. URL http://dx.doi.org/10.1186/1471-2105-12-s8-s5.

C.-H. Wei, H.-Y. Kao, and Z. Lu. Sr4gn: A species recognition software tool for gene normalization. *PLOS ONE*, 7:e38460, 6 2012. URL https://journals.plos.org/plosone/article?id=10.1371/journal.pone.0038460.

C.-H. Wei, H.-Y. Kao, and Z. Lu. Gnormplus: An integrative approach for tagging genes, gene families, and protein domains. *BioMed Research International*, 2015:e918710, 2015. URL https://www.hindawi.com/journals/bmri/2015/918710/.

C.-H. Wei, L. Phan, J. Feltz, R. Maiti, T. Hefferon, and Z. Lu. tmvar 2.0: integrating genomic variant information from literature with dbsnp and clinvar for precision medicine. *Bioinformatics*, 34(1):80–87, 2018.

C.-H. Wei, A. Allot, R. Leaman, and Z. Lu. PubTator central: Automated concept annotation for biomedical full text articles. *Nucleic Acids Research*, 47(W1):W587–W593, 2019.

A. Wen, S. Fu, S. Moon, M. El Wazir, A. Rosenbaum, V. C. Kaggal, S. Liu, S. Sohn, H. Liu, and J. Fan. Desiderata for delivering nlp to accelerate healthcare ai advancement and a mayo clinic nlp-as-a-service implementation. *NPJ digital medicine*, 2(1):130, 2019.

Q. T. Zeng, S. Goryachev, S. Weiss, M. Sordo, S. N. Murphy, and R. Lazarus. Extracting principal diagnosis, co-morbidity and smoking status for asthma research: evaluation of a natural language processing system. *BMC medical informatics and decision making*, 6(1): 1–9, 2006.

Y. Zhang, Y. Zhang, P. Qi, C. D. Manning, and C. P. Langlotz. Biomedical and clinical english model packages for the stanza python NLP library. *Journal of the American Medical Informatics Association*, 28(9):1892–1899, 2021. URL https://doi.org/10.1093/jamia/ocab090.
